# Illness identity perception of adolescents with anxiety and mood disorders and its association with social functioning

**DOI:** 10.3389/fpubh.2025.1713205

**Published:** 2026-01-12

**Authors:** Talia Yemini Gueta, Liron Lamash, Adi Stern

**Affiliations:** 1 Department of Occupational Therapy, Faculty of Social Welfare and Health Sciences, University of Haifa, Haifa, Israel; 2 Department of Occupational Therapy, Faculty of Health Sciences, Ben-Gurion University of the Negev, Beer-Sheva, Israel

**Keywords:** illness identity, adolescence, anxiety, mood disorder, social functioning

## Abstract

**Introduction:**

Adolescence represents a critical period of identity development. Individuals navigate biological maturation and evolving social roles while integrating different self-assets into a coherent sense of self. Illness identity, the degree to which individuals integrate their health condition into their sense of self, significantly affects psychological well-being and social functioning in adolescents with chronic conditions.

**Methods:**

This cross-sectional study examined illness identity profiles in adolescents with anxiety and mood disorders and their associations with social functioning domains. Forty-three adolescents aged 16–18 years (*M* = 16.57, *SD* = 0.72), diagnosed with anxiety or mood disorders, completed an online questionnaire battery, including the Depression Anxiety Stress Scales-Youth version, Illness Identity Questionnaire, Social Emotional Health Survey-Secondary, Youth Engagement and Satisfaction in Social Life Questionnaire, and Inventory of Romantic Relationship Competence.

**Results:**

Analysis revealed no differences between the positive and negative identity dimensions. The Illness Identity Questionnaire total score was negatively correlated with psychological distress (*r* = −0.44, *p* < 0.01) and positively correlated with social-emotional strengths (ρ = 0.30, *p* = 0.05). Although no significant correlations emerged with social engagement measures or romantic relationship competence overall, follow-up analyses revealed specific dimensional associations.

**Discussion:**

This study extends the literature on illness identity by demonstrating its relevance for psychological well-being, social-emotional strengths, and relational functioning among adolescents with anxiety and mood disorders. The findings emphasize that illness identity is a multidimensional construct with adaptive and maladaptive dimensions shaping adolescents' experiences. *Engulfment* emerged as a particular risk factor, whereas *acceptance* and *enrichment* were linked to more positive outcomes. The study highlights the importance of considering identity development in adolescent mental health research and practice.

## 1 Introduction

Adolescence, the transitional period between childhood and adulthood, is generally defined as spanning the ages of 10 to 24 years (1). Biological maturation and significant shifts in social roles characterize this intricate and distinctive developmental period. It entails progression from the immaturity and social dependence of childhood toward the responsibilities and expectations of adult life (1, 2). The overarching objective during adolescence is to achieve developmental potential, identity formation, and social integration (1, 3).

Mental health disorders are highly prevalent in adolescence, with anxiety and mood disorders (AMD) representing the most common conditions (4). Anxiety disorders, affecting 4.4%−5.5% of adolescents, are characterized by excessive fear, worry, and apprehensive expectations accompanied by behavioral disturbances that cause significant distress or impair daily functioning. Together with somatic and cognitive changes that impair overall functioning, persistent sadness, emptiness, or irritability mark depressive disorders, whose prevalence rates reach 1.4%−3.5%—notably higher among older adolescents. Bipolar disorders are less common among adolescents, with estimated prevalence rates ranging from 0.9% to 3.9%. These conditions are characterized by alternating episodes of depression and elevated mood states, such as mania or hypomania (5, 6). A substantial degree of comorbidity exists among these disorders, and their co-occurrence is associated with poorer prognosis and greater functional challenges (7, 8).

Anxiety and mood disorders carry significant social consequences. Adolescents with AMD often experience academic difficulties, emotional dysregulation, and reduced participation with peers, at school, and in community life. These experiences may culminate in withdrawal, marginalization, and social isolation (9, 10). Growing evidence suggests that this process is not one-way but bidirectional: while mental health disorders restrict adolescents' engagement, social isolation intensifies depressive and anxiety symptoms, reinforcing their psychological distress (11, 12). Withdrawal and exclusion experiences have been consistently linked to heightened symptom internalization and decreased well-being (13, 14). This interplay underscores the need for explanatory frameworks beyond symptom counts and clinical categories. Understanding how adolescents perceive and integrate their mental health condition into their self-concept may provide crucial insights into why, despite similar symptom severity, some youth remain socially engaged and others withdraw. Such perspectives are particularly valuable in vulnerable populations, where illness-related perceptions may shape pathways toward social resilience or isolation.

Developing a coherent sense of identity is a central developmental task in adolescence. It guides behavior, decision-making, and self-perception and fosters a sense of continuity and psychological well-being. This process becomes particularly critical as adolescents strive for autonomy and social belonging, making stable identity formation essential for individual adjustment and successful integration into peer and community life (15–17). In the context of chronic conditions, including mental health disorders, the *illness identity* concept refers to the degree to which one integrates a health condition into their overall identity (18, 19).

Oris et al. (19) proposed four dimensions of illness identity: engulfment, rejection, acceptance, and enrichment. *Engulfment* indicates the degree to which a person feels the illness dominates their identity; they may define themselves only in terms of the illness. *Rejection* refers to how much the person rejects the illness as part of their identity and can often take a toll on illness self-management and adherence to treatment. *Acceptance* is the degree to which a person accepts their illness as part of their identity, aside from other self-defining identity assets, without being overwhelmed. *Enrichment* is when a person feels the illness has changed their values, improved their life outlook, and enabled them to grow personally (19, 20). Engulfment and rejection capture rather maladaptive illness identity components, whereas acceptance and enrichment are more adaptive modes of illness integration. These illness identity dimensions are related to physical and psychological functioning across diverse chronic health conditions, including type 1 diabetes (19, 21), celiac disease (22), and neurodevelopmental disorders such as autism (23) or attention deficit hyperactivity disorder (ADHD) (24), making them valuable and clinically significant. Recently, a qualitative study applied this framework to adolescents with internalizing problems (mood, anxiety and/or eating disorders), highlighting the coexistence of multiple illness identity domains and ambivalence between them. Nevertheless, the application of illness identity framework to adolescents with AMD remains relatively underexplored, underscoring the need for further research in this area (25). Given that these conditions are often “hidden” and stigmatized, adolescents may encounter unique challenges in integrating them into their self-concept, with possible implications for social participation and adjustment (26, 27).

Social-emotional strengths are positive psychological assets that support adolescents in understanding and regulating emotions, developing empathy, initiating interpersonal relationships, and coping adaptively with life's challenges. Commonly studied strengths include hope, self-control, optimism, persistence, and the sense of social belonging (28). These strengths are especially important during adolescence because they foster social confidence, peer connection, and a sense of belonging within school and community environments. Research has consistently shown that adolescents with higher levels of such strengths report greater engagement in social and academic contexts, stronger peer relationships, and reduced feelings of loneliness and exclusion (28, 29). Most studies on social-emotional strengths have focused on general adolescent populations. However, recent findings indicate that these strengths may also function as protective factors among adolescents experiencing anxiety and depression. For example, adolescents who report higher levels of hope, optimism, and meaning in life demonstrated reduced levels of anxiety and depression symptoms (30). Illness-related perceptions may be critical in determining whether adolescents with AMD can draw on these internal strengths to support social integration or whether stigma and self-concept challenges hinder their expression. In this context, examining how social–emotional strengths relate to illness identity may offer valuable insights into adolescents' capacity for resilience and social integration.

Whereas social-emotional strengths reflect internal psychological resources, social participation represents the external enactment of these capacities in everyday interactions with family, peers, school, and community. *Social participation* is active involvement in social activities. It encompasses face-to-face and technology-mediated interactions (31–33). This construct overlaps with *social engagement*, which reflects the extent of actual involvement and satisfaction with participation (34, 35). Beyond daily functioning, participation in adolescence is a key developmental milestone strongly associated with belonging, self-esteem, and long-term social integration. Recent evidence shows that adolescents who report a stronger sense of belonging at school and online settings display fewer psychosocial difficulties and better academic outcomes (36). However, participation avoidance of school and social contexts, withdrawal from family and peers, and reduced leisure engagement often restrict participation for adolescents coping with AMD, in turn limiting their role functioning and connectedness (9, 10, 37). Illness identity may shape these trajectories because an adolescent's perception of their diagnosis can determine whether they experience their condition as a barrier to social integration or a manageable part of self that allows continued engagement.

One specific and increasingly salient domain of adolescent social participation is romantic competence. This multifaceted construct comprises various skills and attributes, including conflict resolution, intimacy, prosocial behavior, self-control, emotion regulation, social confidence, initiative, assertiveness, social efficacy, empathy, and sympathy. Engagement in romantic relationships increases during adolescence; some individuals start pursuing long-term committed partnerships. Thus, developing these romantic competence skills contributes positively to social competence and overall adjustment (38). Adolescents with AMD may face particular challenges in this area because their mental health difficulties can affect their self-confidence, emotional availability, and ability to sustain close relationships.

Research on adolescent romantic relationships and depressive symptoms has shown that positive coping and self-perceived friendship competence can protect youth from depressive symptoms related to romantic experiences. Those who cope effectively with daily stressors, especially in romantic contexts, report fewer depression and anxiety symptoms, better social functioning, and higher self-esteem (39). Adolescents with strong emotion-repair abilities and skills in managing and improving negative moods are likelier to develop stable and supportive romantic relationships, marked by higher social competence and more positive interactions (40). Conversely, depressive symptoms during adolescence have been associated with greater conflict and reduced satisfaction in romantic relationships (41). Although this literature underscores the link between romantic functioning and internalizing symptoms (42), romantic competence has not yet been explicitly examined among adolescents with AMD. Understanding how illness identity relates to social participation and romantic competence may provide important insights into the broader social consequences of AMD beyond symptom severity.

Despite growing recognition of the social and emotional challenges faced by youth with AMD (12–15), the role of illness identity in this population remains underexplored. Prior research on illness identity has primarily focused on chronic physical or neurodevelopmental conditions such as diabetes, celiac disease, ADHD, and autism (19, 22–24). These studies demonstrate that adaptive illness identity, marked by acceptance and enrichment, is associated with better psychological adjustment, whereas maladaptive patterns like rejection and engulfment predict distress and poorer functioning. However, the application of this framework to AMD in adolescents remains limited. These conditions are among the most prevalent mental health concerns worldwide, with lifetime rates exceeding 20% (4, 6, 9), yet they often remain invisible and stigmatized. Adolescents coping with AMD must navigate both internal symptoms and external perceptions that may complicate identity formation and social integration (11, 26, 27).

Consequently, a critical gap exists in understanding how adolescents with AMD integrate their diagnosis into their self-concept and how illness identity relates to protective psychological assets, such as social-emotional strengths, or to social domains, including participation and romantic competence. Addressing this gap is particularly relevant in light of this population's risks for social withdrawal and isolation. Building on previous illness identity research, the present study extends this framework to AMD, examining associations between illness identity perception and psychological distress, social-emotional strengths, social engagement, and romantic competence. By situating illness identity within the context of adolescent mental health, this study aims to illuminate developmental processes that may inform more holistic intervention approaches. We hypothesize that:

1. Similar to patterns observed in other clinical populations, adolescents with AMD will report higher levels of positive illness identity dimensions (i.e., acceptance, enrichment) than negative dimensions (i.e., rejection, engulfment).
2. A more positive illness identity perception will be negatively associated with lower levels of psychological distress.
3. A more positive illness identity perception will be positively associated with higher levels of social-emotional strengths.
4. A more positive illness identity perception will be positively associated with greater social engagement in all environments.
5. A more positive illness identity perception will be positively associated with greater romantic competence.

## 2 Materials and methods

### 2.1 Study design and participants

This cross-sectional study used an anonymous online survey. Convenience and snowball sampling were used, which are common and practical approaches in adolescent mental health research where participant recruitment can be challenging. The *a priori* sample size calculation using the G^*^Power Statistical Program (Version 3.1.9) indicated that 84 participants were needed to detect a moderate effect size (*p* = 0.3, α = 0.05, power = 0.80). Eligibility screening required participants to confirm a formal diagnosis of AMD provided by an authorized child and adolescent psychiatrist. Inclusion criteria were adolescents aged 16–18 years and the practical ability to complete the online survey independently (access to the internet and sufficient reading proficiency). We designed the exclusion criteria to minimize confounding conditions independently associated with social functioning difficulties. Specifically, participants with primary psychotic disorders, epilepsy, or autism spectrum disorder were excluded. However, we did not systematically exclude other chronic medical or neurodevelopmental conditions (e.g., ADHD or gastrointestinal conditions); instead, we documented their presence and considered them when interpreting the findings.

The final sample comprised 43 adolescents, reflecting the challenges of recruiting this population. Specifically, adolescents with clinically diagnosed AMD are often reluctant to participate in self-report research due to the topic's sensitive nature and the demands of lengthy questionnaires, a limitation well-documented in prior studies of adolescent mental health surveys (43, 44). The sample included adolescents aged 16 to 18 years (*M* = 16.57, *SD* = 0.72), 31 (72.1%) of whom were identified as female, 11 (25.6%) were identified as male and one (2.3%) was identified as trans male. The mean age at diagnosis was 14.19 years (*SD* = 1.71). Twenty-two adolescents (51.1%) reported having a comorbid ADHD diagnosis. Table 1 presents the sample's demographic and clinical characteristics.

**Table 1:** Demographics and clinical characteristics.

**Characteristic**	***n***	**%**
**Gender**		
Female	31	72.1
Male	11	25.6
Trans male	1	2.3
**Primary diagnosis**		
Anxiety	19	44.2
Depression	7	16.3
Anxiety and depression	16	37.2
Bipolar disorder	1	2.3
**Comorbidity reported**		
Attention deficit hyperactivity disorder	22	51.1
Juvenile idiopathic arthritis	1	2.3
Borderline personality	1	2.3
Tourette syndrome	1	2.3
Gastrointestinal motility disorder	1	2.3
**Treatment status**		
Prescribed medication	29	67.4
Currently taking medication	19	44.2
Taking medication irregularly	2	4.7
Receiving psychotherapy	21	48.8

### 2.2 Procedure

The University of Haifa Institutional Ethics Committee approved this study (approval number 274/19-1). Participants were recruited through advertisements on social media platforms and relevant online forums. Interested participants accessed an online study link containing study information and an informed consent form. Parental consent was waived by the Institutional Ethics Committee, in accordance with international ethical standards, allowing adolescents aged 16–18 to provide their own informed consent for this anonymous, minimal-risk study. After providing consent, participants completed an anonymous questionnaire battery on Google Forms. The data collection took place from December 2024 to April 2025. To safeguard participants' well-being, the researchers also provided contact details and information on youth support services.

### 2.3 Measures

#### 2.3.1 Demographic questionnaire

We designed and used a self-report demographic questionnaire to support the study's inclusion and exclusion criteria and gather general sociodemographic characteristics, such as age, gender, diagnosis, and current treatments.

#### 2.3.2 Depression anxiety stress scales

The Depression Anxiety Stress Scales-Youth version (DASS-Y) (45) was used to assess the degree to which participant's experience general psychological distress symptoms. The DASS-Y is a valid self-report screening questionnaire for adolescents. The DASS-Y includes 21 items rated on a four-point Likert scale from 0 (*not at all*) to 3 (*very much*). Its results offer improved differentiation between depression, anxiety, and stress by providing a comprehensive total score (0–63) and individual scores (0–21) for each of the three subscales. The DASS-Y demonstrates strong psychometric properties among adolescents with AMD, including construct validity supported by confirmatory factor analysis of the three-factor structure, convergent and discriminant validity, and high internal reliability across subscales (α = 0.84–0.89) (45, 46). In our sample, the DASS-Y demonstrated excellent internal consistency (α = 0.90 for the total scale; α = 0.85–0.89 for subscales).

#### 2.3.3 Illness identity questionnaire

The Illness Identity Questionnaire (IIQ) (19) was used to assess the participant's illness identity perception. The IIQ is a self-report questionnaire addressing illness identity perception. It comprises 25 items in the four theoretical identity dimensions: engulfment (eight items), rejection (five items), acceptance (five items), and enrichment (seven items). The first two dimensions (engulfment and rejection) refer to a person's negative perception of their illness identity. The other two (acceptance and enrichment) describe more adaptive illness integration and refer to a positive perception of the illness identity. Participants rate their agreement with each item on a five-point Likert scale from 1 (*strongly disagree*) to 5 (*strongly agree*). An average score is calculated for each dimension; higher scores indicate a higher presence of the specific illness identity dimension. A total score is calculated as the average of all items (after reversing items in the negative components); higher total scores mean more positive illness identity perception (19). The IIQ has demonstrated robust psychometric properties in clinical populations, including good internal consistency (α = 0.75–0.95) and factorial validity (19, 47, 48). For our study, we adapted the IIQ for adolescents with AMD by replacing the term “Illness” with “Anxiety” and or “Mood Disorder,” following the IIQ authors' guidelines (19). This is the first time that the IIQ have been used among adolescents with AMD, therefore there is no psychometric criteria specific to this population. Internal consistency in our sample was acceptable for all items (α = 0.72) and dimensions (α = 0.73–0.88).

#### 2.3.4 Social emotional health survey-secondary

The Social Emotional Health Survey-Secondary (SEHS-S) (28) was used to assess social-emotional strength among participants. The SEHS-S is a 36-item strength-based questionnaire assessing adolescents' social-emotional co-vitality and positive psychological dispositions on a four-point Likert scale from 1 (*not at all true*) to 4 (*very much true*). The measure comprises 12 subscales across four domains: belief in self, belief in others, emotional competence, and engaged living. Scoring involves calculating subscale and domain mean scores and a co-vitality total score, all maintaining the original four-point scale. The SEHS-S has demonstrated strong psychometric properties, including higher-order factor structure, sociocultural and gender invariance, reliability (α = 0.93–0.95 for the co-vitality score), and convergent validity with measures of subjective well-being (28, 49, 50). The SEHS-S has not been specifically utilized in populations diagnosed with AMD. In our sample, it demonstrated excellent internal consistency (α = 0.92 for the total scale; α = 0.78–0.91 for the subscales).

#### 2.3.5 Youth engagement and satisfaction in social life questionnaire

The Youth Engagement and Satisfaction in Social Life (YES-Life) (Stern, Lamash, 2024, unpublished) was used to assess participants' engagement level in various social domains and their satisfaction with it. The YES-LIFE is a new questionnaire developed to assess adolescents' social engagement levels in different social domains: core family life, wider family life, school life, afterschool life, and peer and civic engagement, and their satisfaction with their engagement in these domains. It includes 32 statements describing everyday situations requiring social interaction in various aspects. Adolescents rate each statement for their levels of involvement and satisfaction with their involvement on a five-point Likert scale from 1 (*very small extent*) to 5 (*very large extent*). They then mark whether they wish for a change in that domain (*yes/no*) and what that change might be (*being more involved*/*less involved*). Mean scores are calculated for each social domain and for the total score; higher scores indicate higher engagement and satisfaction levels. The YES-Life was developed with content validity established through expert focus groups and adolescent feedback. No additional forms of validity have been reported. To date, the YES-Life has been used among adolescents with ADHD, demonstrating high internal reliability within these populations (α = 0.96–0.98) (24). In the current sample, the YES-Life demonstrated excellent internal consistency (α = 0.92 for the engagement scale; α = 0.89 for the satisfaction scale) and sufficient to good reliability across the domains (α = 0.78–0.88).

#### 2.3.6 Inventory of romantic relationship competence

The Inventory of Romantic Relationship Competence (IRRC) (38) was used to assess participants' romantic relationship competence. The IRRC is a 35-item self-report assessment that measures adolescents' and young adults' perceptions of their romantic relationship competence, regardless of their experience with relationships. Participants rate their agreement with each item on a five-point Likert scale from 1 (*almost never/never true*) to 5 (*almost always/always true*); higher scores indicate greater perceived competence in each domain. In addition to the total score, the items are divided into seven domains: perspective taking, emotion regulation, conflict resolution skills, intimacy openness, temperament, romantic appeal, and relationship maintenance. The IRRC demonstrates good reliability (α = 0.75–0.93) and strong psychometric properties, including convergent validity with measures of social competence (*r* = 0.44–0.47) and self-esteem (*r* = 0.26–0.46), and divergent validity from unrelated measures (38). The IRRC has not been specifically utilized in populations diagnosed with AMD. In our sample, the IRRC demonstrated good internal consistency (α = 0.81 for the total scale; α = 0.51–0.85 for subscales).

#### 2.3.7 Statistical analysis

Before conducting statistical analyses, we manually examined the dataset for response quality, specifically to detect unusual or inconsistent answering patterns (e.g., identical responses across multiple items). We identified no indications of unreliable or invalid data and retained all cases for analysis. We conducted the data analysis using IBM SPSS (Version 27.0). Descriptive statistics (frequencies, ranges, means, and standard deviations) were used to describe the sample and study variables. Cronbach's alpha coefficients were calculated to assess the internal consistency of all questionnaires in the current sample. One-way repeated measures analysis of variance (ANOVA) with the Bonferroni correction was used to examine the differences between the four IIQ dimensions (hypothesis 1). Pearson's correlation coefficients (marked as *r*) were applied for variables that demonstrated approximately normal distributions, and Spearman's correlation coefficients (marked as ρ) were applied for variables that demonstrated non-normal distributions. These coefficients were used to examine the associations between illness identity (IIQ) and psychological distress (DASS-Y; hypothesis 2), social-emotional strengths (SEHS-S; hypothesis 3), social engagement and satisfaction (YES-Life; hypothesis 4), and romantic competence (IRRC; hypothesis 5).

## 3 Results

### 3.1 Descriptive statistics of study measures

Descriptive statistics (ranges, means, and standard deviations) and internal reliabilities (Cronbach's Alpha) for all study measures in the current sample are presented in Table 2.

**Table 2:** Descriptive statistics and internal reliability of the IIQ, DASS-Y, SEHS-S, YES-Life and IRRC.

**Variables**	**Range**	**M**	**SD**	**Cronbach's Alpha**
**IIQ**				
Rejection	1.20-4.60	2.90	0.86	0.76
Acceptance	1.00-5.00	3.40	0.91	0.73
Engulfment	1.25–5.00	3.39	0.87	0.88
Enrichment	1.57–4.86	3.22	0.78	0.74
Total score	2.28–3.68	3.03	0.42	0.72
**DASS-Y**				
Depression	0.00–21.00	10.44	6.22	0.89
Anxiety	0.00–21.00	9.90	5.92	0.85
Stress	0.00–21.00	12.98	5.17	0.85
Total score	0.00–58.00	33.33	13.47	0.90
**SEHS-S**				
Belief-in-self	1.00–3.78	2.49	0.63	0.78
Belief-in-others	1.00–3.89	2.82	0.63	0.78
Emotional competence	1.00–4.00	3.07	0.59	0.85
Engaged living	1.00–3.78	2.05	0.73	0.91
Total score	1.00–3.58	2.61	0.50	0.92
**YES-life engagement**				
Core family	1.00–4.71	3.07	0.91	0.82
Wide family	1.00–5.00	2.75	0.99	0.84
Peers in school	1.00–4.60	2.57	0.95	0.77
Peers after-school	1.00–4.40	2.48	0.88	0.82
Civic life	1.00–3.50	1.80	0.82	0.60
Total engagement score	1.00–3.88	2.59	0.72	0.92
**YES-life satisfaction**				
Core family	1.14–4.57	3.28	0.85	0.80
Wide family	1.00–5.00	3.16	1.00	0.85
Peers in school	1.00–5.00	3.17	0.91	0.77
Peers after-school	1.00–5.00	3.17	0.79	0.80
Civic life	1.00–5.00	3.00	1.17	0.83
Total satisfaction score	1.63–4.31	3.17	0.63	0.89
**IRRC**				
Locus of control	1.88–5.00	3.27	0.78	0.82
Perspective taking	1.83–5.00	4.01	0.78	0.85
Romantic appeal	1.00–5.00	2.48	0.95	0.51
Intimacy openness	1.00–4.20	2.40	0.96	0.80
Emotion regulation	1.25–5.00	2.65	1.04	0.85
Temperament	1.33–5.00	2.99	0.95	0.60
Conflict resolution	2.17–4.33	3.13	0.61	0.67
Total score	2.43–4.49	3.09	0.46	0.81

IIQ, illness identity questionnaire; DASS-Y, depression anxiety stress scales-youth version; SEHS-S, social emotional health survey-secondary; YES-Life, youth engagement and satisfaction in social life; IRRC, inventory of romantic relationship competence.

### 3.2 Illness identity dimensions

**Figure 1:**
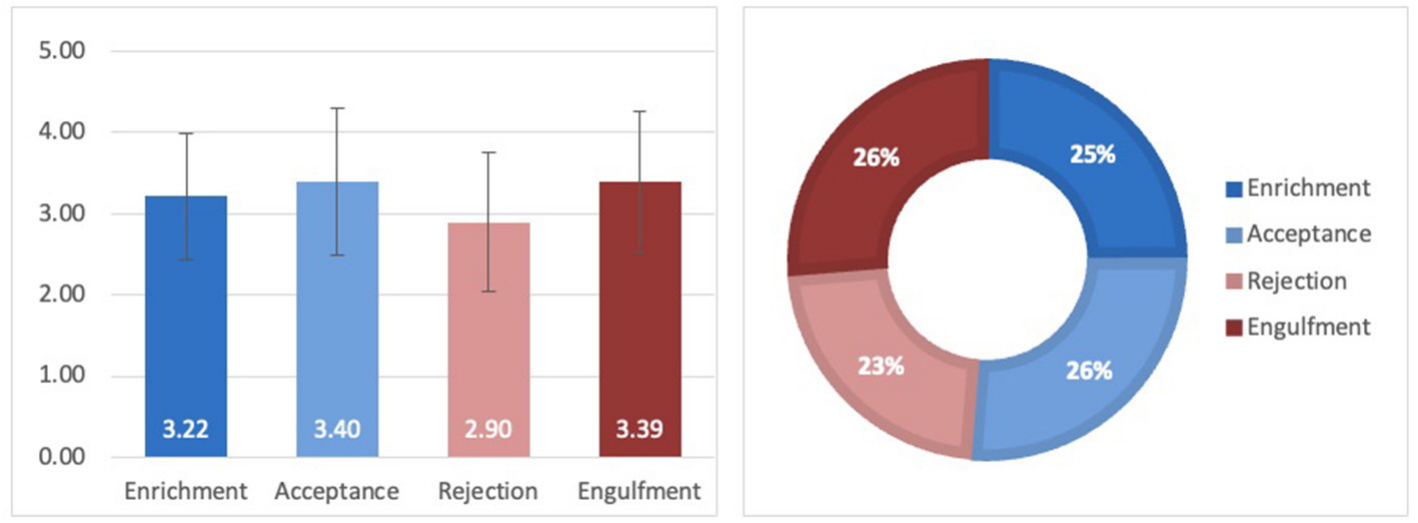
Mean scores, standard deviations, and distribution of the illness identity dimensions.

Our results indicated a significant overall difference among the illness identity dimensions, *F*(3, 126) = 3.29, *p* < 0.05, partial η^2^ = 0.073. However, follow-up pairwise comparisons with Bonferroni correction did not reach statistical significance (Figure 1). Therefore, the first hypothesis was not supported, as no significant differences were found between the illness identity dimensions, suggesting a relatively balanced illness identity perception across positive and negative dimensions.

A multiple linear regression was conducted to examine whether gender and ADHD predicted IIQ total scores. The overall model was not significant, *F*(2, 40) = 1.63, *p* = 0.209, explaining 7.5% of the variance (adjusted *R*^2^ = 0.029). Neither gender (β = −0.06, *p* = 0.679) nor ADHD (β = 0.27, *p* = 0.081) emerged as significant predictors. Variance inflation factors (VIFs < 1.02) indicated no multicollinearity. These findings confirm that ADHD symptoms did not act as a confounding variable in this sample.

### 3.3 Correlations between illness identity and psychological distress

Supporting hypothesis 2, a more positive illness identity was associated with lower psychological distress. The IIQ total score showed a moderate negative correlation with the DASS-Y total score (*r* = −0.44, *p* < 0.01). Specifically, adolescents who reported a more positive perception of their illness identity also reported experiencing fewer symptoms of psychological distress. Conversely, higher levels of distress were associated with a more negative illness identity. Follow-up analyses focusing on the IIQ dimensions revealed that the engulfment dimension was strongly and consistently related to psychological distress. Specifically, higher engulfment was associated with greater depressive symptoms (*r* = 0.53, *p* < 0.001), anxiety (*r* = 0.43, *p* = 0.001), and stress (*r* = 0.51, *p* < 0.001). These findings suggest that adolescents who perceive their disorder as overwhelming or dominating their identity are more likely to experience elevated levels of emotional difficulties; conversely, higher levels of emotional difficulties may contribute to perceiving the disorder as more central or overwhelming. We found no significant associations between the other illness identity dimensions (rejection, acceptance, or enrichment) and the DASS-Y subscales.

### 3.4 Correlations between illness identity and social–emotional strengths

In line with hypothesis 3, a positive correlation of moderate magnitude was found between the IIQ and SEHS-S total scores (ρ = 0.30, *p* = 0.05), indicating that adolescents with a more positive illness identity tended to report stronger social–emotional assets. Follow-up analyses of the IIQ dimensions and SEHS-S subscales revealed several notable patterns, with higher engulfment consistently associated with lower self-efficacy, self-awareness, and self-belief. In contrast, enrichment was positively related to self-efficacy and self-awareness. In addition, rejection showed a negative correlation with peer support, and acceptance was negatively related to gratitude. Table 3 presents the full set of correlations.

**Table 3:** Correlations between illness identity questionnaire (IIQ) dimensions and social emotional health survey-secondary (SEHS-S) subscales.

**SEHS-S**	**IIQ**				
	**Rejection**	**Acceptance**	**Engulfment**	**Enrichment**	**Total score**
Self-efficacy	0.14	**−0.35** ^*^	**−0.52** ^***^	**0.32** ^*^	0.30
Self-awareness	0.25	−0.06	**−0.38** ^*^	**0.35** ^*^	**0.31** ^*^
Persistence	**−0.32** ^*^	−0.05	−0.22	−0.01	0.25
Belief-in-self subscale	0.02	−0.19	**−0.49** ^***^	0.29	**0.38** ^**^
School support	0.03	0.05	−0.00	0.08	0.07
Family support	0.03	0.23	−0.20	0.05	0.20
Peer support	**−0.43** ^*^	0.15	0.03	0.00	0.22
Belief-in-others subscale	–0.17	0.20	–0.07	0.05	0.23
Emotional regulation	0.17	−0.01	−0.22	0.19	0.19
Empathy	**0.32** ^*^	0.28	0.10	0.04	−0.07
Self-control	**0.34** ^*^	−0.06	−0.04	0.22	0.01
Emotional competence subscale	**0.35** ^*^	0.01	−0.08	0.19	0.04
Optimism	0.30	−0.25	−0.12	0.27	−0.01
Gratitude	0.03	**−0.45** ^**^	**−0.54** ^***^	0.19	0.25
Zest	0.08	−0.04	−0.13	**0.31** ^*^	0.20
Engaged living subscale	0.16	−0.27	−0.29	0.30	0.16
Co-vitality total score	0.06	−0.03	−0.29	0.26	**0.30** ^*^

^*^p < 0.05; ^**^p < 0.01; ^***^p < 0.001. Bold values indicate statistically significant correlations.

### 3.5 Correlations between illness identity and social engagement

Hypothesis 4 was not supported, as no significant associations were found between the IIQ total score and the YES-Life social engagement or satisfaction indices. Follow-up analyses of the illness identity dimensions revealed one significant finding: higher enrichment was associated with lower wider family engagement (*r* = −0.34, *p* < 0.05). Although not statistically significant, a positive association emerged between acceptance and wider family engagement (*r* = 0.29, *p* = 0.06). We observed no other significant correlations between the illness identity dimensions and social engagement domains.

### 3.6 Correlations between illness identity and romantic relationship competence

We found no significant correlation between the IIQ and IRRC total scores. However, follow-up analyses of the IIQ dimensions and IRRC revealed several significant associations. Higher enrichment was positively associated with better temperament (*r* = 0.34, *p* < 0.05). Higher rejection was negatively associated with intimacy openness (*r* = −0.41, *p* < 0.01), and higher engulfment was linked to lower intimacy openness (*r* = −0.44, *p* < 0.01) and poorer conflict resolution (*r* = −0.43, *p* < 0.01). Therefore, hypothesis was partially supported.

## 4 Discussion

This study extends the literature on illness identity by demonstrating its relevance for adolescents with AMD and by contributing to the emerging understanding of mental illness identity in adolescents. As this study used a cross-sectional design, the observed relationships should be interpreted as correlational rather than causal. The results shed light on how illness identity is linked to not only psychological distress but also protective assets and social functioning.

Contrary to our hypothesis, the findings did not support the expectation that adolescents with AMD would show more adaptive than maladaptive identity components, as observed in other populations. Adolescents with celiac disease demonstrated an illness identity profile that was more positive and adaptive (both acceptance and enrichment) than negative (22) and adolescents with ADHD (24) and autism reported significantly higher acceptance scores compared to other illness identity dimensions (23). One possible explanation for this divergence is that AMD differs from these conditions. Unlike chronic physical illnesses such as celiac disease, adolescents with AMD may not view their diagnosis as permanent and central to their identity and therefore may not experience clearly defined positive or negative feelings toward it. It is also possible that adolescents with AMD face greater stigma (25) than those with celiac disease, despite both conditions interfering with daily functioning, which may inhibit adaptive identity processes. Furthermore, unlike ADHD and ASD, which involve outwardly observable behaviors and frequent social feedback, AMD is largely internal and less visible. Research on invisible health conditions suggests that reduced visibility provides fewer identity shaping cues, potentially leading adolescents to experience the positive and negative dimensions of illness identity in a more balanced way (51). Moreover, since most prior studies have focused on chronic physical or externally noticeable conditions, internalizing conditions like AMD remain understudied (52). The present findings therefore offer a novel perspective by showing that illness identity may develop differently when the condition is not outwardly evident. Nevertheless, the relatively balanced illness identity profile across the four domains found in the current study, aligns with findings from a recent qualitative study of adolescents with internalizing problems (25). This emphasize that adaptive and maladaptive dimensions may coexist, depending on the context of each adolescent's life. This balanced profile highlights the multidimensional nature of illness identity in adolescents with AMD and supports the need for longitudinal studies to capture possible shifts over time.

Our study found that a more positive illness identity perception was associated with lower psychological distress levels, supporting hypothesis 2. At the same time, this relationship appears to operate in the opposite direction as well, such that more negative illness identity perceptions are linked to higher levels of depression and anxiety. Consistently, engulfment stood out as the dimension most strongly linked with depression, anxiety, and stress. This pattern may reflect a process in which the illness defines the adolescent's overall identity, contributing to heightened distress and potentially indicating internalized stigma (“I am my illness”). These results are consistent with broader identity research showing that positively correlated identity confusion with internalizing symptoms in adolescence (53). Similar associations have also been observed in adolescents with chronic illnesses, with negative self-evaluations contributing to higher depression rates (13). Notably, engulfment has been specifically identified as a predictor of poorer psychological functioning (21), supporting the notion that maladaptive outcomes are more likely when the illness dominates self-perception. Importantly, these associations may be bidirectional: maladaptive identity perceptions like engulfment may be associated with vulnerability to distress, whereas high depression or stress levels may be linked to stronger feelings of engulfment, making it harder for adolescents to separate their diagnosis from their overall self-concept (54, 55). This bidirectional pattern may offer a novel contribution by illustrating how identity processes and emotional distress are linked to each other in adolescents with internalizing conditions such as AMD. Recognizing this reciprocal influence underscores the need for further research to examine possible causal pathways and clinical interventions to address identity integration and emotional well-being simultaneously.

As hypothesized, positive illness identity was associated with greater social-emotional strengths, particularly self-efficacy, self-awareness, and empathy. This finding aligns with previous studies using the SEHS-S in different adolescent populations, which consistently demonstrated that higher co-vitality predicts lower psychological distress and enhances well-being in youth (28, 56, 57). Moreover, it is in line with broader identity-development research linking a coherent and adaptive sense of self to better mental health outcomes and identity confusion to greater emotional difficulties (58). Furthermore, longitudinal evidence suggests that identity processes and emotional strengths may influence each other over time, with bidirectional associations documented between identity development and internalizing symptoms (54, 55). Together, these results suggest that adaptive illness identity is not only linked to lower distress but is also associated with greater psychological resilience, offering new evidence on how illness identity relates to co-vitality in adolescents with AMD and the importance of identity-focused, strengths-based interventions for this population.

As opposed to the fourth hypothesis, illness identity showed no significant correlations with overall social engagement or satisfaction. At the domain level, the engagement level with wider family alone was negatively associated with enrichment feelings. These limited associations suggest that illness identity may not directly determine adolescents' overall participation, echoing findings in autism that linked identity dimensions more strongly to quality of life than to actual engagement (59). It may further indicate that additional factors beyond illness identity, such as family dynamics, educational environments, and social resources, influence social participation. The specific pattern with wider family may reflect the complexities of disclosure and expectations. Adolescents who experience their diagnosis as a source of growth (enrichment) may invest more heavily in peer and school contexts, where autonomy and mutual understanding are greater, and less in extended family relationships, which can be shaped by stigma or ambivalence. Similar associations between adaptive illness identity and social participation have been reported in celiac disease (22), suggesting that context and diagnosis may moderate these dynamics. Importantly, the direction of influence may operate both ways: social participation experiences in the wider family could support acceptance or, when negative, strengthen rejection and engulfment. Although our findings did not reveal significant associations between illness identity and social participation, recent work suggests that mental illness identity is shaped by a combination of individual and contextual factors (such as stigma, support, and perceptions of the illness), which can influence adolescents' social experiences in different ways (25). This highlights the need for further research to examine how these processes operate specifically in internalizing conditions such as AMD.

Finally, hypothesis 5 was partially supported, as illness identity was not associated with overall romantic competence, but specific patterns emerged across the IIQ domains. Enrichment was associated with a more adaptive relational temperament, rejection was related to intimacy avoidance, and higher levels of engulfment were linked to poorer conflict resolution skills and intimacy avoidance. These specific associations are consistent with recent findings showing that self-stigma and negative illness perceptions can foster interpersonal withdrawal and reduced emotional openness, thereby limiting adolescents' capacity to engage in close and supportive romantic interactions (60). Likewise, difficulties in conflict resolution associated with engulfment align with evidence that heightened emotional distress and rigid self-focus interfere with effective communication and increase vulnerability to interpersonal conflict in close relationships (61). In contrast, enrichment may reflect a more coherent and growth-oriented self-perception, which is associated with greater emotional maturity, better relational functioning, and healthier romantic dynamics during adolescence (62). Taken together, these results indicate that illness identity dimensions may shape specific relational processes, such as intimacy, communication, and conflict management, rather than global romantic competence, underscoring the importance of further research on romantic development in adolescents with internalizing conditions such as AMD.

### 4.1 Clinical implications

This study's findings carry several clinical implications for the intervention and support of adolescents with AMD. Mental health assessment has increasingly emphasized both positive and negative dimensions, recognizing that well-being and distress must be considered together (57, 63, 64). Moreover, positive mental health, including self-acceptance, supportive relationships, and life purpose, predicts recovery from mood disorders (65–67). In line with this perspective, our findings underscore the need to expand the assessment of adolescents with AMD beyond symptoms; it should include illness identity, social-emotional strengths, and various aspects of social participation. Addressing how adolescents perceive their diagnosis and engage in relationships may guide more tailored interventions to strengthen social-emotional resources and foster adaptive identity development (68, 69). At the same time, these implications should be interpreted cautiously, as the cross-sectional design prevents drawing causal conclusions about the directionality of these associations.

The consistent association between engulfment and psychological distress highlights the importance of addressing maladaptive illness identity processes in therapeutic settings. Clinicians may help adolescents differentiate between their diagnosis and their broader sense of self, reducing the risk of adolescents defining themselves primarily through their symptoms. Cognitive-behavioral and narrative approaches and psychoeducation for families can provide tools to support this separation. Moreover, the links between positive illness identity dimensions and social-emotional strengths suggest that interventions should not only target symptom reduction but also foster acceptance and enrichment. Strengths-based approaches, such as positive psychology interventions and social-emotional learning frameworks, may enhance self-efficacy, empathy, and resilience, thereby buffering against distress and supporting developmental progression. However, these potential intervention targets should be viewed as plausible mechanisms rather than confirmed pathways, given that the associations observed do not establish causality. Although no strong associations emerged between illness identity and overall social engagement, the patterns observed with wider family relationships point to the role of context in shaping identity. Interventions that involve families, particularly in addressing stigma, disclosure, and support, could be valuable for promoting healthier engagement and reinforcing acceptance. Finally, the associations between illness identity and specific romantic competence aspects underline the need to consider relational functioning as part of adolescent mental health care. Supporting the development of conflict resolution, intimacy, and emotion regulation skills may improve relationship outcomes and contribute to more adaptive identity integration. Therefore, integrating modules on peer and romantic relationships within existing therapeutic programs may have a dual benefit for social development and identity formation.

### 4.2 Limitations and future directions

This study has several limitations that should be considered when interpreting its findings. Its cross-sectional design prevents conclusions about causality. Although illness identity was linked to psychological distress, social-emotional strengths, and aspects of romantic competence, the direction of these associations cannot be determined. Longitudinal designs are needed to clarify whether maladaptive identity feelings are associated with greater vulnerability to distress and social challenges or whether these difficulties, in turn, are linked to subsequent increases in maladaptive identity dimensions.

The study's reliance on convenience and snowball sampling may have introduced selection bias, as participants were likely those more accessible or connected within certain networks. Consequently, the sample may not fully represent the broader adolescent population, which could limit the generalizability of the findings. Future research should consider employing more diverse or probabilistic sampling methods to enhance representativeness. In addition, the study relied solely on adolescent self-report questionnaires, which may be influenced by social desirability bias, mood state, or limited self-awareness. Incorporating parent, teacher, or clinician perspectives and observational or behavioral measures could provide a more comprehensive understanding of illness identity and functioning. Future studies should incorporate mixed methods approach to provide a more comprehensive understanding of illness identity and psychosocial functioning.

The final sample size was smaller than the recommended sample size from our *a priori* power analysis, which may have reduced the statistical power and limited the ability to detect significant effects. This should be considered when interpreting the results. Moreover, the modest sample size (although adequate for the analyses conducted), convenience-based recruitment, and predominance of female participants limit the generalizability of the findings. The presence of ADHD symptoms in our sample was high (51.1%), exceeding the general population average (70). However, these elevated rates of ADHD comorbidity among adolescents with AMD have been previously reported (71). Larger and more diverse samples would allow for stronger conclusions and subgroup analyses. Finally, the study collected basic demographic information. Thus, continues studies should include additional information such as illness duration and severity, treatment type and history, and educational setting, which may shape illness identity and psychosocial adjustment.

Future studies should examine these factors to better understand the contexts in which adaptive or maladaptive illness identity develops. Future research should also move to consider person-centered methods that capture distinct illness identity profiles. Longitudinal and intervention studies will be particularly valuable for clarifying the reciprocal influences between identity, psychological functioning, and social development. Such research could inform tailored clinical interventions that address both identity integration and broader well-being in adolescents with AMD.

## 5 Conclusion

In summary, this study extends the literature on illness identity by demonstrating its relevance for psychological well-being, social-emotional strengths, and relational functioning among adolescents with AMD. The findings emphasize that illness identity is a multidimensional construct, with adaptive and maladaptive dimensions linked to distinct patterns of social experiences. Engulfment emerged as a potential risk factor, whereas acceptance and enrichment were related to more positive outcomes. In turn, the patterns observed may also be bidirectional, as social experiences may shape identity development. While these relationships are correlational and do not imply causality, they underscore the importance of considering identity development in adolescent mental health research and practice. Supporting adolescents in integrating their diagnosis into a broader and more balanced sense of self may serve as a promising direction for future interventions aimed at promoting resilience, participation, and relational competence.

## Data Availability

The raw data supporting the conclusions of this article will be made available by the authors, without undue reservation.
